# Predicting Changes in Depression Severity Using the PSYCHE-D (Prediction of Severity Change-Depression) Model Involving Person-Generated Health Data: Longitudinal Case-Control Observational Study

**DOI:** 10.2196/34148

**Published:** 2022-03-25

**Authors:** Mariko Makhmutova, Raghu Kainkaryam, Marta Ferreira, Jae Min, Martin Jaggi, Ieuan Clay

**Affiliations:** 1 École Polytechnique Fédérale de Lausanne Lausanne Switzerland; 2 Evidation Health Inc San Mateo, CA United States; 3 Digital Medicine Society Boston, MA United States

**Keywords:** depression, machine learning, person-generated health data

## Abstract

**Background:**

In 2017, an estimated 17.3 million adults in the United States experienced at least one major depressive episode, with 35% of them not receiving any treatment. Underdiagnosis of depression has been attributed to many reasons, including stigma surrounding mental health, limited access to medical care, and barriers due to cost.

**Objective:**

This study aimed to determine if low-burden personal health solutions, leveraging person-generated health data (PGHD), could represent a possible way to increase engagement and improve outcomes.

**Methods:**

Here, we present the development of PSYCHE-D (Prediction of Severity Change-Depression), a predictive model developed using PGHD from more than 4000 individuals, which forecasts the long-term increase in depression severity. PSYCHE-D uses a 2-phase approach. The first phase supplements self-reports with intermediate generated labels, and the second phase predicts changing status over a 3-month period, up to 2 months in advance. The 2 phases are implemented as a single pipeline in order to eliminate data leakage and ensure results are generalizable.

**Results:**

PSYCHE-D is composed of 2 Light Gradient Boosting Machine (LightGBM) algorithm–based classifiers that use a range of PGHD input features, including objective activity and sleep, self-reported changes in lifestyle and medication, and generated intermediate observations of depression status. The approach generalizes to previously unseen participants to detect an increase in depression severity over a 3-month interval, with a sensitivity of 55.4% and a specificity of 65.3%, nearly tripling sensitivity while maintaining specificity when compared with a random model.

**Conclusions:**

These results demonstrate that low-burden PGHD can be the basis of accurate and timely warnings that an individual’s mental health may be deteriorating. We hope this work will serve as a basis for improved engagement and treatment of individuals experiencing depression.

## Introduction

Major depressive disorder is a leading cause of disability worldwide, impacting the lives of more than 264 million people globally, according to the World Health Organization [1]. The COVID-19 pandemic has further increased the number of people experiencing depressive symptoms [2]. Despite its prevalence, depression often remains undiagnosed and untreated. In 2017, an estimated 17.3 million adults in the United States experienced at least one major depressive episode, with 35% of them not receiving any treatment [3].

Underdiagnosis of depression has been attributed to many reasons, including stigma surrounding mental health, limited access to medical care, and barriers due to cost [4]. Undiagnosed and untreated depression has significant economic consequences, adding an economic burden of over US $200 billion annually in the United States alone [5]. Thus, it is essential to make the detection and monitoring of depression symptoms easier and more affordable.

An increasingly explored and promising way to accomplish this is through person-generated health data (PGHD) in the form of self-reports and data from consumer-grade wearable devices [6]. Multiple studies have shown that early indicators of changes in depression status can be detected from PGHD in the form of social media use [7] or physical activity patterns [8]. For example, a recent study, using consumer wearable devices to track the sleep of 368 participants, found several strong associations (Z-scores up to 6.19) between sleep features and self-reported depression [9]. Another study showed that activity features collected for 23 participants could accurately (*κ*=0.773) classify individuals with depression from controls, and predict changes in depression status over a 2-week period [10]. Although these studies are limited in sample size and time duration to generalize across larger populations, they demonstrate the potential of this approach versus more burdensome active assessments [11].

**Figure 1:**
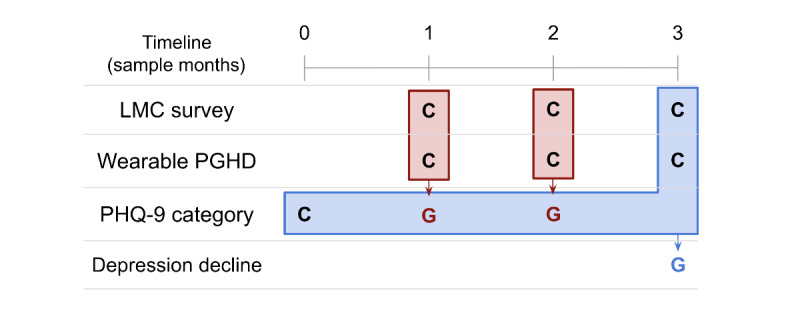
A schematic overview of the PSYCHE-D (Prediction of Severity Change-Depression) model. Phase 1c uses screener survey responses (regarding sociodemographics and chronic comorbidities at baseline), self-reported lifestyle and medication changes (LMC) survey data from the month in which the Patient Health Questionnaire-9 (PHQ-9) label is generated, and data from consumer-grade wearables to categorize each individual’s likely PHQ-9 category. In the second phase, this generated information is combined with the initial PHQ-9 category, screener survey responses, additional LMC self-reports, and consumer-grade wearable device person-generated health data (PGHD) to make the final prediction of whether the individual is likely to have experienced increased depression severity over the 3-month period. Red blocks represent Phase 1, and blue blocks represent Phase 2. C: collected. G: generated.

In this work, we present PSYCHE-D (Prediction of Severity Change-Depression), a 2-phase prediction model that uses PGHD to predict longitudinal changes in an individual’s depression severity level (Figure 1). Input data include self-reported sociodemographic data and medical history, and objective behavioral data derived from consumer-grade wearables. The presented model has been developed using the largest longitudinal cohort study ever considered for depression at the time of publication [12], collecting PGHD over a 1-year period from more than 10,000 participants.

In previous work, we presented initial results [13] for the first phase of the model, and exploratory results for the second phase are also available [14]. These initial results demonstrate the feasibility of the PSYCHE-D approach, yet the stepwise development approach creates the possibility of data leakage between the phases and therefore misleading results. This work presents results from an improved pipeline that eliminates the leakage, thus ensuring generalizable results and laying the foundation for a very low–burden, consumer-facing, personalized system that could improve engagement and outcomes in people with depression.

## Methods

### Data Collection

The data used in this work are part of the DiSCover (Digital Signals in Chronic Pain) Project (ClinicalTrials.gov identifier: NCT03421223). The DiSCover Project is a 1-year longitudinal study consisting of 10,036 individuals in the United States, who, between January 2018 and January 2020, provided data from consumer-grade wearable devices and completed surveys about their mental health and lifestyle changes quarterly and monthly, respectively. Detailed design and baseline participant characteristics are described in the report by Lee et al [12].

The data subset used in this work comprises the following:

1. Wearable PGHD: Step and sleep data from the participants’ consumer-grade wearable devices (Fitbit) worn throughout the study were collected.
2. Screener survey: Prior to the study, participant self-reported sociodemographic information, as well as comorbidities were collected.
3. Lifestyle and medication changes (LMC) survey: Every month, participants were requested to complete a brief survey reporting changes in their lifestyle and medication over the past month.
4. Patient Health Questionnaire-9 (PHQ-9) score: Every 3 months, participants were requested to complete the PHQ-9, a 9-item questionnaire that has proven to be a reliable and valid measure of depression severity [15].

From these input sources, we defined a range of input features, both static (defined once, remain constant for all samples from a given participant throughout the study, eg, demographic features) and dynamic (varying with time for a given participant, eg, behavioral features derived from consumer-grade wearables). Feature extraction and engineering are described in Multimedia Appendix 1.

### Data Processing

#### Data Filtering Process

**Figure 2:**
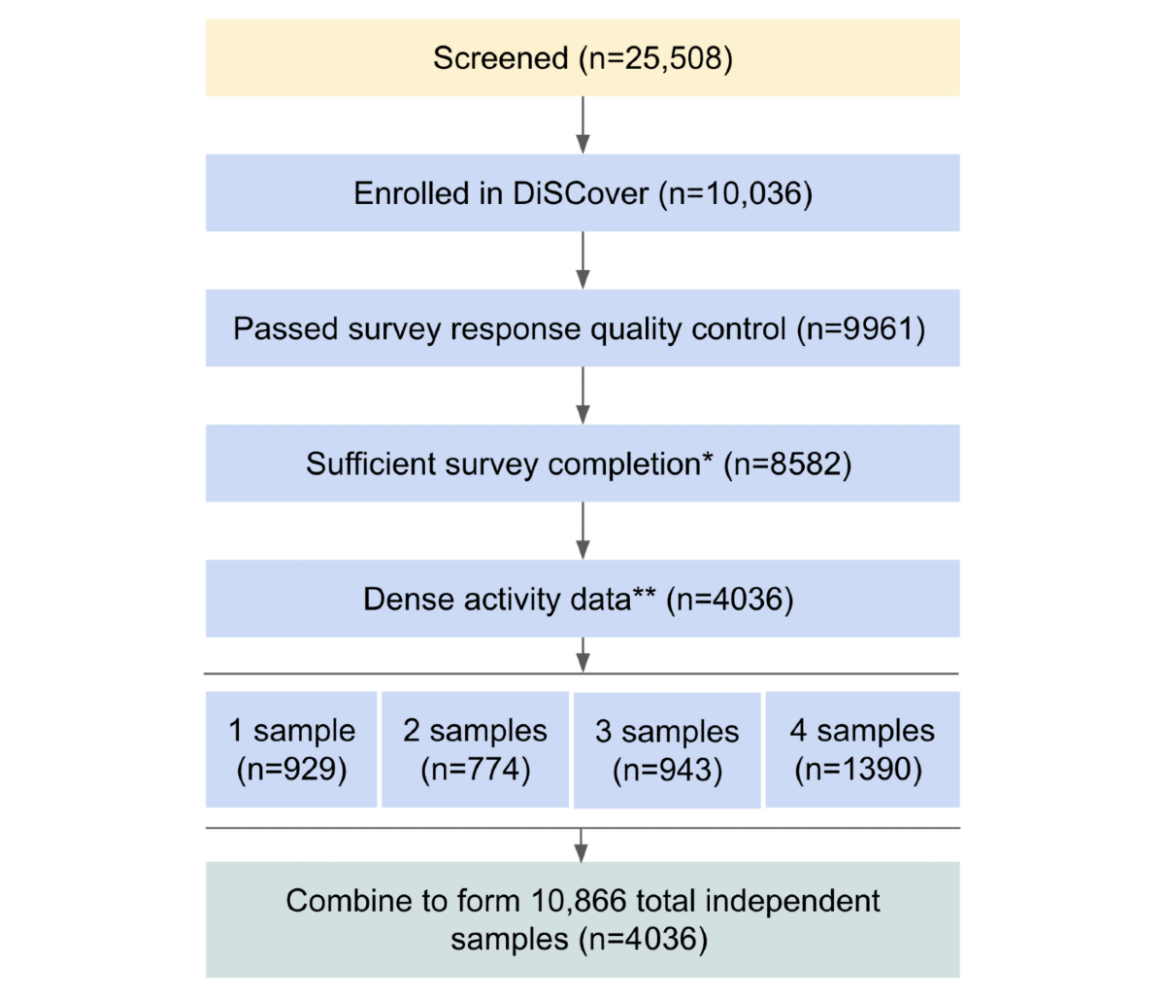
Illustration of the participant filtering process. *Completion of the Patient Health Questionnaire-9 (PHQ-9) for the current quarter, the PHQ-9 for the previous quarter, and the lifestyle and medication changes (LMC) survey for the current month. **≥10 hours daily wear time for ≥4 days per week in the 2-week interval. DiSCover: Digital Signals in Chronic Pain.

Figure 2 outlines the processing of the initial data set into the samples used for developing the phase 1c model. Of the 10,036 enrolled participants, 9961 passed the survey response quality control, defined as completion of the PHQ-9 for at least two contiguous quarters, as well as the LMC survey for the same month as the second PHQ-9. Additional filtering, based on the density of available activity data in the 2 weeks matching the PHQ-9 recall period, was performed according to standards proposed in the literature [16,17]. We ultimately obtained a total of 10,866 samples from 4036 unique participants.

#### Data Set Construction

Initial data exploration showed that the evolution of PHQ-9 scores over 3-month intervals was constant throughout the study period, when grouping by demographic variables, such as sex, age, race, and geographic location. Based on this observation, we divided the data into 3-month long, nonoverlapping, independent samples. We used the notations “SM0” (sample month 0), “SM1,” “SM2,” and “SM3” to refer to relative time points within each sample. Each 3-month sample consisted of 1 set of screener survey responses, PHQ-9 survey responses at SM0 and SM3, LMC survey responses at SM3 (as well as SM1 and SM2, if available), and wearable PGHD for SM3 (as well as SM1 and SM2, if available). The wearable PGHD included data collected from 8 to 14 days prior to the PHQ-9 label generation date (SM1 and SM2 in phase 1, SM3 in phase 2).

### Modeling

**Figure 3:**
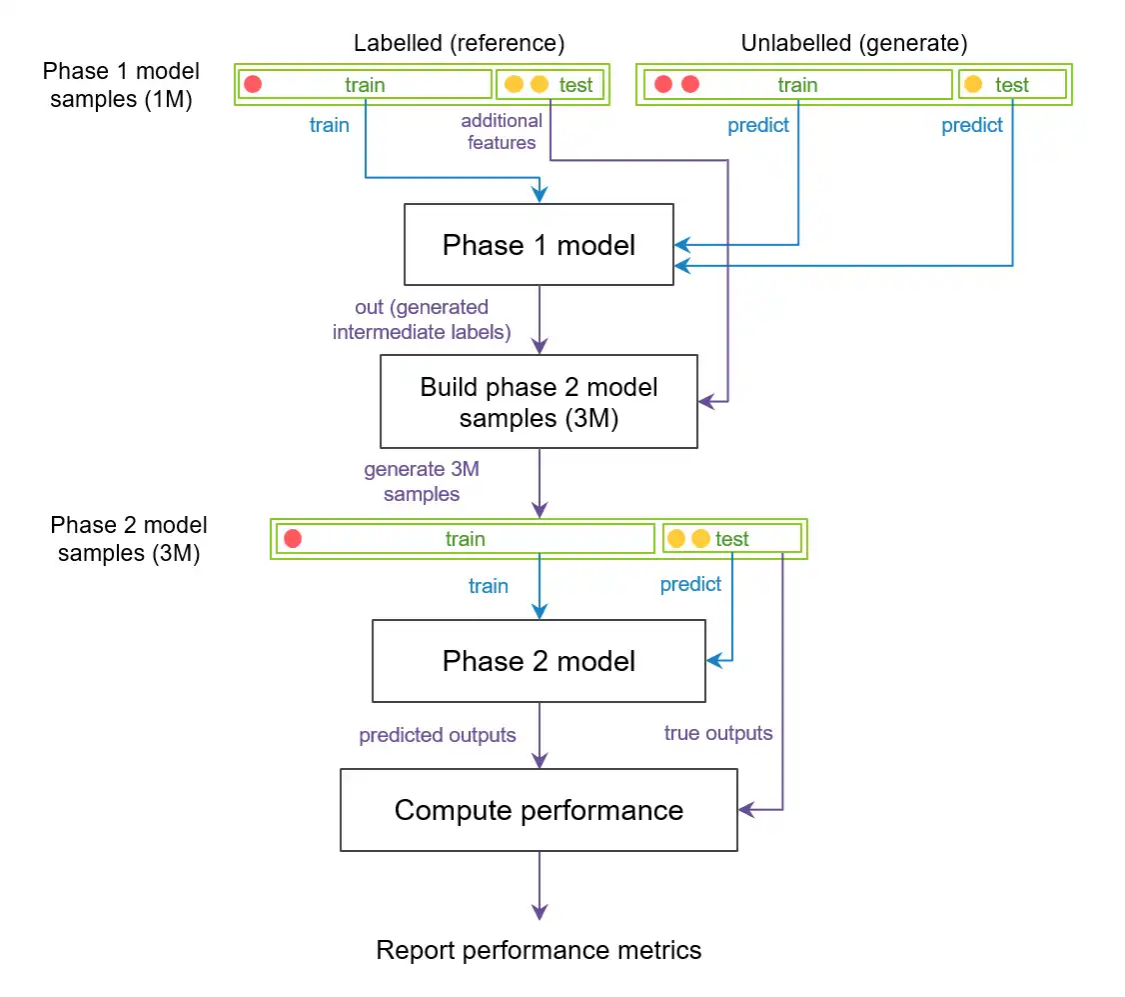
Schematic representation of the PSYCHE-D (Prediction of Severity Change-Depression) combined pipeline architecture, used to estimate performance on previously unseen participants. The phase 1c model is trained on a subset of participants in the training set, and predictions for the training and test set participants are made. The phase 2c model has the same participant split for the training and test sets. Red and yellow circles represent samples from 2 different participants. All samples from the red participant are in the training set, and all samples from the yellow participant are in the test set for both phases 1c and 2c. Green blocks represent data, and black blocks represent models and data processing stages. Blue arrows represent input to classification models for training or predicting, and purple arrows represent data passage for other purposes (eg, providing true output values for testing). Note: multiple circles represent multiple samples from the same participant. This procedure is repeated over 5 random participant-based splits of the training and test data, to obtain confidence intervals for the combined pipeline performance.

Figure 1 illustrates the overall approach, and the inputs and outputs for phase 1c and phase 2c. Figure 3 illustrates the modeling approach, which is explained in more detail below. The key design feature is that the models are combined into a single combined pipeline, and participant-based train-test partitioning is performed once at the start, in order to eliminate the possibility of data leakage. The combined pipeline is thus fitted on 1 set of participants and tested on another set of previously unseen participants.

#### Phase 1c: Categorization of the Intermediate PHQ-9

The goal of the phase 1c model was to predict participants’ PHQ-9 score categories from sociodemographic, medical, and wearable PGHD. The initial version of phase 1c is described in the report by Makhmutova et al [13]. Here, we describe an improved variant that has been adapted to reduce overfitting. The Light Gradient Boosting Machine (LightGBM) algorithm with Dropouts Meet Multiple Additive Regression Trees (DART) boosting [18], an ensemble model of boosted regression trees with dropout, was chosen due to its ability to handle sparse data and the ability to tune an additional dropout parameter to reduce overfitting. Feature selection removed highly correlated features, and used recursive feature elimination [19] in order to eliminate features that had lower contributions to model performance. Model performance was primarily measured using quadratic weighted Cohen *κ* [20], with adjacent accuracy (ie, fraction of samples predicted at most one off from the target value), balanced accuracy, and weighted F1-scores as secondary performance metrics. We performed randomized search 5-fold cross-validation to tune the hyperparameters of our LightGBM model. We chose to perform a 5-fold cross-validation to reduce the impact of overfitting. We reported the performance metrics of the best tuned models with 95% CIs across 5 training runs (5 outer shuffle splits). Further details on hyperparameters are reported in Multimedia Appendix 1 and elsewhere [14]. Due to the very large feature space that covers a range of static and dynamic input features, we constructed the model in 3 steps. We first performed an extensive exploration accessing the best feature subsets of each type of input. We then carried out an initial optimization on input sets, which combined different types of input and considered an initial estimation of model error. Subsequently, we conducted a final tuning to obtain the best performing model. The output of phase 1c generated intermediate monthly PHQ-9 score categories for SM1, denoting sample month 1, and SM2.

#### Phase 2c: Prediction of Longitudinal PHQ-9 Change

In phase 2c, we predicted an increase in the PHQ-9 category using the participants’ PHQ-9 scores from SM0, intermediate generated PHQ-9 categories at SM1 and SM2 as well as the generated probabilities of each PHQ-9 category for SM1 and SM2, and LMC survey responses and wearable PGHD collected over the 2 weeks prior to final PHQ-9 completion at SM3. We also used the screener survey responses as input features to control for sociodemographic factors. To compute the target variable in each sample in the phase 2c model, we observed whether there was an increase in the PHQ-9 category between SM0 and SM3.

A similar model construction procedure was used for phase 2c as for phase 1c. The feature selection procedure consisted of reducing the initial number of input features through the removal of highly correlated features and selecting the most important features using recursive feature elimination with cross-validation for the largest sets of input features, grouped by source. Then, we performed forward sequential feature selection [21], a greedy method that has been successfully used to develop digital measures in mental health studies [22], to identify the optimal features. We then again used LightGBM DART, as it has been shown to deliver high accuracy in comparable classification tasks [18], is able to handle sparse data, and generates interpretable models.

Specificity and area under the precision-recall curve (AUPRC) [23] were prioritized as performance metrics. Feature importance was assessed using a combination of the following 2 key metrics: “gain” importance and “split” importance [24]. Gain importance measures the improvement in accuracy that a feature provides, while split importance considers the number of times the feature is used in a model. Taken together, these metrics help us understand which features contribute the most to the “decisions” that the model makes.

The construction of the PSYCHE-D combined pipeline consisting of phase 1c, followed by phase 2c, is summarized in Figure 3. The diagram also illustrates the participant-based splitting approach used to ensure that we generate predictions on previously unseen participants, to evaluate the approach’s generalization capabilities. Further details are presented in Multimedia Appendix 1.

### Code Availability

The codes of the models in this study, along with their trained weights, are available on GitHub [25].

### Data Availability

Data are made available to academic researchers on Zenodo [26].

### Ethics Approval

This study received expedited review and Institutional Review Board (IRB) approval from the Western Institutional Review Board-Copernicus Group (IRB study number: 1181760; protocol number: 20172916; initial approval date: December 21, 2017).

## Results

### Overview

In the following section, we present the performance and informative features for the combined pipeline. Importantly, we wanted to build the model in a manner representative of how such a model might be deployed “in the real world.” In such a situation, a trained model (eg, as part of an app) would need to make predictions for participants that the model is naive to, that is, people who have just downloaded the app and perhaps only filled out the baseline assessments, and did not contribute data used in the model construction. This pipeline is therefore designed to test the generalizability of the models by eliminating any data leakage, and using a participant-based validation strategy, that is, the model is tested on participants that it is completely naive to. Results for the 2 phases are presented separately.

### Intermediate Classification of Depression Severity

Acquiring PGHD on a large scale requires a low-burden data collection approach; thus, participants were only asked to complete the PHQ-9 at sparse intervals, once every 3 months. Consequently, we were limited to a relatively small set of reference labels, with 2.07 labels on average per enrolled participant over the course of 1 year. The first phase of our approach thus generated more frequent intermediate depression severity labels, which were used in combination with self-reported reference labels to reduce the sparsity of the data set by up to 3 times.

We were able to construct a multi-class classification model that determines a participant’s depression severity for a given month, by assigning an individual to 1 of 5 ordinal PHQ-9 classes describing severity from minimal to severe [15]. The details and distributions of the observed classes are presented in Table S1 in Multimedia Appendix 1.

The best performing model, based on the LightGBM DART algorithm, after hyperparameter tuning, had a *κ* value of 0.476 (95% CI ±0.017) and an adjacent accuracy of 77.6%.

**Figure 4:**
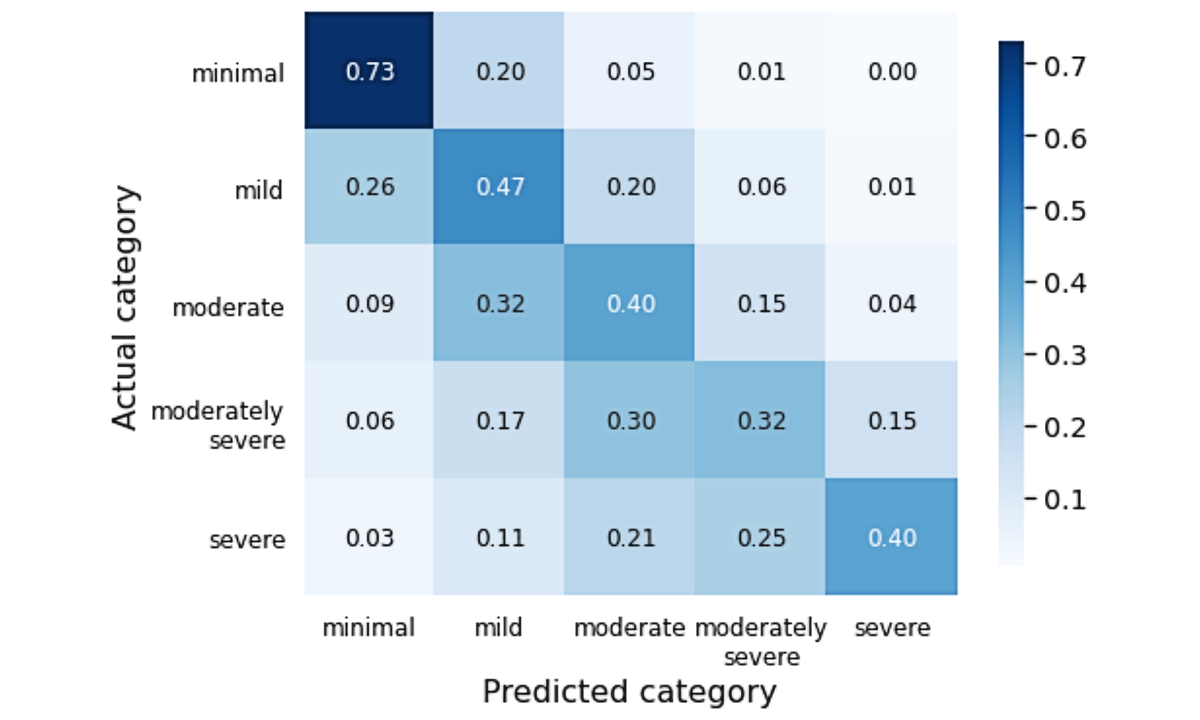
Confusion matrix showing the phase 1c model’s Patient Health Questionnaire-9 (PHQ-9) score category accuracy distribution across PHQ-9 severity groups. Darker blue represents higher accuracy. Performance overall is weak, but adjacent accuracy is high, and classification performance in samples from individuals with lower (minimal to mild) and higher (moderately severe to severe) severity is relatively high, compared to the performance seen for intermediate severity samples.

The performance of the model was not equal across all PHQ-9 severity categories. Comparing actual to predicted categories in a confusion matrix (Figure 4), we observed that performance was high for samples from individuals with either relatively low (minimal or mild) or high (moderately severe or severe) depression.

The model included features selected across all input sources, including demographics (gender, birth year, education, and BMI), life events and conditions at baseline (whether they had received financial assistance, experienced trauma or given birth, or were diagnosed with a range of chronic conditions), LMC (changes to medications or lifestyle), and sleep-related wearable PGHD (the number of hypersomnia nights, range of bedtime, and average ratio of the time spent asleep to the time spent in bed). A full list of the final features and their relative importance is included in Multimedia Appendix 2. This model was then used to generate intermediate PHQ-9 category labels for each individual for SM1 and SM2.

### Prediction of Longitudinal Change

The intermediate generation of depression severity labels means that each sample consisted of the PHQ-9 depression severity at SM0, the LMC surveys, wearable PGHD, and up to two generated labels that provide a weak estimate of depression severity (PHQ-9 category) at SM1 and SM2.

We posed our original aim as a binary problem as follows: can we predict increased depression severity? We defined increased depression severity as that when a participant changed the PHQ-9 category between SM0 and SM3. From our 10,866 samples, 2252 (20.7%) were thus labeled as positive cases.

The construction of the second phase model was optimized across possible input feature sets and LightGBM model hyperparameters. As summarized in Figure 3, we noted that with this approach, the optimization process also depended on the outputs generated by the first phase.

We used a range of metrics to assess performance, but prioritized sensitivity as the key metric, as our primary goal in this work was to correctly identify the highest proportion of individuals reporting increased depression severity. As the data set was highly imbalanced, with 21% of individuals in the data set reporting increased depression severity, we optimized for performance for both the majority and minority classes. We thus took into account specificity and AUPRC as secondary performance metrics, to observe the tradeoff in performance for each class.

Based on this, the best performing model selected 13 input features, with a sensitivity of 55.4% (95% CI ±0.8%), specificity of 65.3% (95% CI ±4.2%), and AUPRC of 0.31 (95% CI ±0.024). In comparison, a baseline model, which randomly assigned 20.7% positive labels, reported an AUPRC of 0.21, a sensitivity of 19.8%, and a specificity of 80.0% (averaged across 10 runs of 1000 samples).

We examined the most important features in the second phase of the combined pipeline and observed that the selected features to predict relative changes in depression were similar to the features selected to predict absolute depression in the first phase.

**Figure 5:**
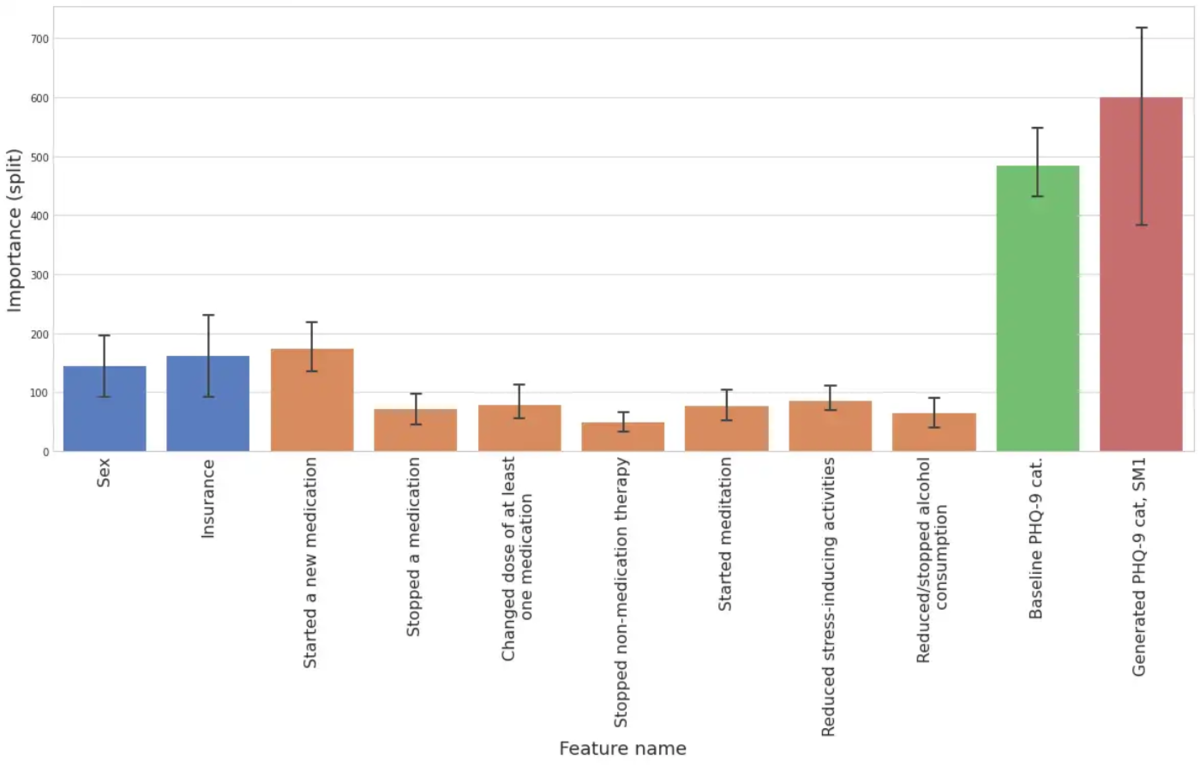
Split feature importance of the features selected in at least three of five train-test splits in the best performing phase 2c model. Colors represent the types of features (static screener features are blue, lifestyle and medication changes [LMC] features are orange, baseline Patient Health Questionnaire-9 [PHQ-9] features are green, and generated PHQ-9 features are red). The 95% CIs for the split feature importance are also visualized. SM: sample month.

The most important features are presented in Figure 5, with further details in Multimedia Appendix 2. Features that were most frequently selected as strong predictors of an increase in depression severity, regardless of the cohort, were PHQ-9–related features. Specifically, the self-reported starting PHQ-9 category and the generated intermediate PHQ-9 category for SM1 were the most important features, as we can see in Figure 5. Among the static demographic and socioeconomic features, we noted that sex and having health insurance were the most important. Various self-reported LMC features were frequently selected, including medication changes (starting, stopping, and changing doses) and stress-related lifestyle changes (starting meditation and reducing stress-inducing activities), as well as reducing or stopping alcohol consumption. We observed that objective sleep features were again selected, but no specific individual wearable PGHD feature (sleep or otherwise) was sufficiently consistently selected to be included in the final model.

## Discussion

### Principal Findings

PGHD represent a low burden direct connection to the patient journey, and such data have already been demonstrated to be a valuable component of models that predict health-relevant outcomes [27,28]. We present a 2-phase approach for predicting longitudinal deterioration in depression status. In phase 1c, we increased the label density by generating intermediate PHQ-9 category labels using wearable PGHD and LMC information. In the second phase, we combined self-reported and generated PHQ-9 category labels with additional recent wearable PGHD and LMC information to predict the deterioration of depression status 3 months after the initial self-report. This 2-phase approach has a very low burden and requires very little participant interaction. The information we used as input consists of simple self-reports and data from consumer-grade wearables.

Even though overall performance in phase 1c was not particularly strong (*κ*=0.476, 95% CI ±0.017), we were encouraged by 2 factors: the adjacent accuracy was high (77.6%), and an examination of features in the final tuned models showed good correspondence to factors known to be important risk factors for depression, for example, gender, experience of trauma, and chronic comorbidities. Large-scale studies have shown that these have an influence on depression [29]. We also observed that objective sleep features were selected. Sleep features and depressive disorders have been previously associated using low-cost wearable devices [9], PGHD [30], and smartphones [31]. Additionally, we observed that performance was not even across severity groups and was high for individuals with either relatively mild or relatively severe depression.

In phase 2c, our best performing model achieved a sensitivity of 55.4%, specificity of 65.3% (95% CI ±4.2%), and AUPRC of 0.31 (95% CI ±0.024). In comparison, simulating random assignment of 20.7% positive labels across 10 iterations of 1000 samples, we noted an AUPRC of 0.21, a sensitivity of 19.8%, and a specificity of 80.0%. This means that sensitivity nearly tripled, while specificity only slightly reduced. We prioritized sensitivity because the potential consequences of false negatives (ie, not identifying a person with deteriorating depression) is much higher than the cost of false positives (ie, incorrectly suspecting someone of deteriorating depression).

We observed that features from all input sources were selected in the best performing models, but with different relative importance. We saw that static features (ie, those defined at enrollment, which do not change afterwards) were selected, but were of relatively low importance. This included features that are known to be relevant to the risk of developing depression, including the presence of chronic comorbidities [32], ethnicity [33], financial difficulties [34], and pregnancy [35]. We also saw features derived from wearable devices, including trends in sleep onset time, percentage of sleep time spent awake, and overall number of hypersomnia days. The most important features were those generated in phase 1c, that is, the probability of an individual being in a given PHQ-9 class, summarizing features from across all input sources. The intermediate labels generated in phase 1c are inspired by the concept of “weak labeling,” which can help reduce large-scale noisy data to a signal useful for supervised learning (eg, the report by Zhan et al [36]). We noted that due to data sparsity, intermediate labeling was not always available, and thus, some samples did not have 2 intermediate PHQ-9 category labels, but sometimes had 1 or none. Nonetheless, as LightGBM was able to deal with missing values, the lack of intermediate labeling or missing PGHD values did not pose problems in the phase 2c model predictions, highlighting that the approach described in our work is indeed low-burden and robust.

From this, we were able to deduce that the average sleep onset time is a good determinant of increasing depression severity, which is consistent with previous research [9], but that variability in sleep is participant specific and not necessarily a good predictor for generalizing to other participants.

### Limitations

The work presented here demonstrates the potential of a PGHD-based model for predicting long-term changes in depression status in new individuals. This initial approach nevertheless has several limitations in practice, which will be addressed in future work.

The model relies on the completion of several self-reported surveys over time. Participants were highly engaged with the year-long research study, but to lower the barrier to participation, the number of surveys could be reduced or replaced with alternative sources of data. For example, instead of LMC surveys, medication change data could be obtained through electronic health records [37] or through other consumer-grade wearables that incorporate engagement, such as the Oura ring, which allows participants to annotate days with a number of tags like medication [38].

The performance of PSYCHE-D was below our initial expectations, despite more than triple sensitivity versus a random model, and was weaker than the initial nongeneralized performance [13,14]. However, further validation and prospective data collection could seek to build off this “out of the box” performance using an active learning approach to improve individualized performance [39,40]. We also plan to perform further validation with independently generated data [41]. The study design also limits us to making predictions of depression status change over a 3-month time window. Thus, future work will focus on testing predictions beyond that time horizon.

We will also explore the application of PSYCHE to other aspects of mental health like anxiety [31], fatigue [42], and stress [22].

### Outlook and Conclusion

Effective treatments for depression exist, but they must be delivered in a timely manner, as the benefits of early intervention are established for both older [43] and younger [44] patients. Moreover, the objectivity of our system provides a nonstigmatizing environment to engage people about depression [4]. We hope that this demonstration of the ability to predict long-term changes in depression using a low-burden PGHD-based approach will have great potential to deliver value to patients.

## Appendix

Multimedia Appendix 1 Supplementary methods.

Multimedia Appendix 2 Selected features.

## Abbreviations

AUPRC: area under the precision-recall curve
DART: Dropouts Meet Multiple Additive Regression Trees
DiSCover: Digital Signals in Chronic Pain
IRB: Institutional Review Board
LightGBM: Light Gradient Boosting Machine
LMC: lifestyle and medication changes
PGHD: person-generated health data
PHQ-9: Patient Health Questionnaire-9
PSYCHE-D: Prediction of Severity Change-Depression
SM: sample month

## References

[1] (2021). Depression. World Health Organization.

[2] Ettman CK, Abdalla SM, Cohen GH, Sampson L, Vivier PM, Galea S (2020). Prevalence of Depression Symptoms in US Adults Before and During the COVID-19 Pandemic. JAMA Netw Open.

[3] Major Depression. National Institute of Mental Health.

[4] Chekroud AM, Foster D, Zheutlin AB, Gerhard DM, Roy B, Koutsouleris N, Chandra A, Esposti MD, Subramanyan G, Gueorguieva R, Paulus M, Krystal JH (2018). Predicting Barriers to Treatment for Depression in a U.S. National Sample: A Cross-Sectional, Proof-of-Concept Study. Psychiatr Serv.

[5] Greenberg PE, Fournier A, Sisitsky T, Pike CT, Kessler RC (2015). The economic burden of adults with major depressive disorder in the United States (2005 and 2010). J Clin Psychiatry.

[6] Pedrelli P, Fedor S, Ghandeharioun A, Howe E, Ionescu DF, Bhathena D, Fisher LB, Cusin C, Nyer M, Yeung A, Sangermano L, Mischoulon D, Alpert JE, Picard RW (2020). Monitoring Changes in Depression Severity Using Wearable and Mobile Sensors. Front Psychiatry.

[7] Vahedi Z, Zannella L (2019). The association between self-reported depressive symptoms and the use of social networking sites (SNS): A meta-analysis. Curr Psychol.

[8] Renn BN, Pratap A, Atkins DC, Mooney SD, Areán PA (2018). Smartphone-Based Passive Assessment of Mobility in Depression: Challenges and Opportunities. Ment Health Phys Act.

[9] Zhang Y, Folarin AA, Sun S, Cummins N, Bendayan R, Ranjan Y, Rashid Z, Conde P, Stewart C, Laiou P, Matcham F, White KM, Lamers F, Siddi S, Simblett S, Myin-Germeys I, Rintala A, Wykes T, Haro JM, Penninx BW, Narayan VA, Hotopf M, Dobson RJ, RADAR-CNS Consortium (2021). Relationship Between Major Depression Symptom Severity and Sleep Collected Using a Wristband Wearable Device: Multicenter Longitudinal Observational Study. JMIR Mhealth Uhealth.

[10] Jacobson NC, Weingarden H, Wilhelm S (2019). Digital biomarkers of mood disorders and symptom change. NPJ Digit Med.

[11] Sverdlov O, Curcic J, Hannesdottir K, Gou L, De Luca V, Ambrosetti F, Zhang B, Praestgaard J, Vallejo V, Dolman A, Gomez-Mancilla B, Biliouris K, Deurinck M, Cormack F, Anderson JJ, Bott NT, Peremen Z, Issachar G, Laufer O, Joachim D, Jagesar RR, Jongs N, Kas MJ, Zhuparris A, Zuiker R, Recourt K, Zuilhof Z, Cha J, Jacobs GE (2021). A Study of Novel Exploratory Tools, Digital Technologies, and Central Nervous System Biomarkers to Characterize Unipolar Depression. Front Psychiatry.

[12] Lee J, Cerrada C, Vang M, Scherer K, Tai C, Tran J, Juusola J, Sang C (2021). The DiSCover Project: Protocol and Baseline Characteristics of a Decentralized Digital Study Assessing Chronic Pain Outcomes and Behavioral Data. medRxiv.

[13] Makhmutova M, Kainkaryam R, Ferreira M, Min J, Jaggi M, Clay I (2021). Prediction of self-reported depression scores using person-generated health data from a virtual 1-year mental health observational study. DigiBiom '21: Proceedings of the 2021 Workshop on Future of Digital Biomarkers.

[14] Makhmutova M (2021). Predicting changes in depression using person-generated health data. École Polytechnique Fédérale de Lausanne.

[15] Kroenke K, Spitzer RL, Williams JB (2001). The PHQ-9: validity of a brief depression severity measure. J Gen Intern Med.

[16] Migueles JH, Cadenas-Sanchez C, Ekelund U, Delisle Nyström C, Mora-Gonzalez J, Löf M, Labayen I, Ruiz JR, Ortega FB (2017). Accelerometer Data Collection and Processing Criteria to Assess Physical Activity and Other Outcomes: A Systematic Review and Practical Considerations. Sports Med.

[17] Tudor-Locke C, Camhi SM, Troiano RP (2012). A catalog of rules, variables, and definitions applied to accelerometer data in the National Health and Nutrition Examination Survey, 2003-2006. Prev Chronic Dis.

[18] Korlakai VR, Gilad-Bachrach R (2015). DART: Dropouts meet Multiple Additive Regression Trees. Proceedings of the Eighteenth International Conference on Artificial Intelligence and Statistics.

[19] Guyon I, Weston J, Barnhill S, Vapnik V (2002). Gene Selection for Cancer Classification using Support Vector Machines. Machine Learning.

[20] Fleiss JL, Cohen J (2016). The Equivalence of Weighted Kappa and the Intraclass Correlation Coefficient as Measures of Reliability. Educational and Psychological Measurement.

[21] Aha D, Bankert R, Fisher D, Lenz H (1996). A Comparative Evaluation of Sequential Feature Selection Algorithms. Learning from Data. Lecture Notes in Statistics, vol 112.

[22] Sano A, Taylor S, McHill A, Phillips A, Barger L, Klerman E, Picard R (2018). Identifying Objective Physiological Markers and Modifiable Behaviors for Self-Reported Stress and Mental Health Status Using Wearable Sensors and Mobile Phones: Observational Study. J Med Internet Res.

[23] Saito T, Rehmsmeier M (2015). The precision-recall plot is more informative than the ROC plot when evaluating binary classifiers on imbalanced datasets. PLoS One.

[24] Breiman L (2001). Random Forests. Machine Learning.

[25] PSYCHE-D GitHub open-source repository. GitHub.

[26] Makhmutova M, Kainkaryam R, Ferreira M, Min J, Jaggi M, Clay I (2022). PSYCHE-D: predicting change in depression severity using person-generated health data. JMIR mHealth and uHealth (forthcoming).

[27] Karas M, Marinsek N, Goldhahn J, Foschini L, Ramirez E, Clay I (2020). Predicting Subjective Recovery from Lower Limb Surgery Using Consumer Wearables. Digit Biomark.

[28] Dunn J, Kidzinski L, Runge R, Witt D, Hicks JL, Schüssler-Fiorenza Rose SM, Li X, Bahmani A, Delp SL, Hastie T, Snyder MP (2021). Wearable sensors enable personalized predictions of clinical laboratory measurements. Nat Med.

[29] Brailean A, Curtis J, Davis K, Dregan A, Hotopf M (2020). Characteristics, comorbidities, and correlates of atypical depression: evidence from the UK Biobank Mental Health Survey. Psychol Med.

[30] Kumar S, Tran J, Ramirez E, Lee W, Foschini L, Juusola J (2020). Design, Recruitment, and Baseline Characteristics of a Virtual 1-Year Mental Health Study on Behavioral Data and Health Outcomes: Observational Study. JMIR Ment Health.

[31] Moshe I, Terhorst Y, Opoku Asare K, Sander LB, Ferreira D, Baumeister H, Mohr DC, Pulkki-Råback L (2021). Predicting Symptoms of Depression and Anxiety Using Smartphone and Wearable Data. Front Psychiatry.

[32] Li H, Ge S, Greene B, Dunbar-Jacob J (2019). Depression in the context of chronic diseases in the United States and China. Int J Nurs Sci.

[33] Bailey RK, Mokonogho J, Kumar A (2019). Racial and ethnic differences in depression: current perspectives. Neuropsychiatr Dis Treat.

[34] Richardson T, Elliott P, Roberts R, Jansen M (2017). A Longitudinal Study of Financial Difficulties and Mental Health in a National Sample of British Undergraduate Students. Community Ment Health J.

[35] O'Connor E, Senger CA, Henninger ML, Coppola E, Gaynes BN (2019). Interventions to Prevent Perinatal Depression: Evidence Report and Systematic Review for the US Preventive Services Task Force. JAMA.

[36] Zhan A, Mohan S, Tarolli C, Schneider RB, Adams JL, Sharma S, Elson MJ, Spear KL, Glidden AM, Little MA, Terzis A, Dorsey ER, Saria S (2018). Using Smartphones and Machine Learning to Quantify Parkinson Disease Severity: The Mobile Parkinson Disease Score. JAMA Neurol.

[37] Kukafka R, Ancker JS, Chan C, Chelico J, Khan S, Mortoti S, Natarajan K, Presley K, Stephens K (2007). Redesigning electronic health record systems to support public health. J Biomed Inform.

[38] Ōura Ring company homepage. Ōura Ring.

[39] Huang S, Jin R, Zhou Z (2014). Active Learning by Querying Informative and Representative Examples. IEEE Trans Pattern Anal Mach Intell.

[40] Bachman P, Sordoni A, Trischler A (2017). Learning Algorithms for Active Learning. Proceedings of the 34th International Conference on Machine Learning.

[41] Goldsack JC, Dowling AV, Samuelson D, Patrick-Lake B, Clay I (2021). Evaluation, Acceptance, and Qualification of Digital Measures: From Proof of Concept to Endpoint. Digit Biomark.

[42] Luo H, Lee P, Clay I, Jaggi M, De Luca V (2020). Assessment of Fatigue Using Wearable Sensors: A Pilot Study. Digit Biomark.

[43] Reynolds CF, Cuijpers P, Patel V, Cohen A, Dias A, Chowdhary N, Okereke OI, Dew MA, Anderson SJ, Mazumdar S, Lotrich F, Albert SM (2012). Early intervention to reduce the global health and economic burden of major depression in older adults. Annu Rev Public Health.

[44] Schley C, Pace N, Mann R, McKenzie C, McRoberts A, Parker A (2019). The headspace Brief Interventions Clinic: Increasing timely access to effective treatments for young people with early signs of mental health problems. Early Interv Psychiatry.

