# Supplementary material for: Predicting Changes in Depression Severity Using the PSYCHE-D (Prediction of Severity Change-Depression) Model Involving Person-Generated Health Data: Longitudinal Case-Control Observational Study
Source: JMIR Mhealth Uhealth. 2022 Mar 25;10(3):e34148. doi: 10.2196/34148 (PMC8994145; doi:10.2196/34148)
Supplement: Multimedia Appendix 1 [file mhealth_v10i3e34148_app1.docx]

## **Multimedia Appendix 1.** Supplementary methods.

### Data collection

The data used in this work are part of the DiSCover Project developed by Evidation Health (ClinicalTrials.gov Identifier: NCT03421223; [[1,2]](https://paperpile.com/c/FQjwBF/Phv8x+JHzyd)). The DiSCover Project is a 1-year long longitudinal study consisting of 10,036 individuals who wore consumer wearable devices throughout the study and completed monthly surveys about their mental health and/or lifestyle changes.

More specifically, the data subset used in this work comprises the following:

- *Wearable PGHD*: step and sleep data from the participants’ consumer-grade wearable devices (Fitbit) worn throughout the study
- *Screener survey*: prior to the study, participants were requested to respond to screening and baseline surveys regarding socio-demographic information, as well as comorbidities
- *Lifestyle and medication changes (LMC) survey*: every month, participants were requested to complete a survey reporting changes in their lifestyle and medication over the past month (e.g. changes in eating habits or activity levels, starting new medication)
- *Patient Health Questionnaire (PHQ-9) score*: every 3 months, participants were requested to complete the PHQ-9, a 9-item questionnaire that has proven to be reliable and valid to measure depression severity[[3]](https://paperpile.com/c/FQjwBF/q2lge), in order to track changes in their depression levels

The total age range of participants is 18-85 years, the majority of participants in the cohort are young and middle-aged adults (mean=36.8 years; SD=10.5), 80.5% of the participants are Non-Hispanic White, and 73.7% are female. During the screening stage, participants were asked about pre-existing chronic conditions. There is a high prevalence (58%) of participants reporting chronic pain conditions including migraines, osteoarthritis, fibromyalgia and peripheral nerve pain. In further analysis, we do not differentiate between participants that have been diagnosed with chronic pain conditions and those who haven’t, as participants that report not having been diagnosed with chronic pain conditions may still have underlying conditions.

Figure 4 describes the data collection timeline. At the beginning of the study, participants completed the screener/baseline survey and the PHQ-9. For months 1 through 12, participants were asked to complete the LMC survey documenting their lifestyle and medication changes over the past month. At months 3, 6, 9 and 12 participants were additionally asked to complete the PHQ-9.

The goal of the first phase of PSYCHE-D is to predict depression severity levels, and the goal of the second phase is to predict increase in depression severity. We used the five predefined PHQ-9 score categories[[3]](https://paperpile.com/c/FQjwBF/q2lge), ranging from minimal to severe to measure depression severity. As the PHQ-9 score aims to summarize depression severity over the past two weeks, we only include wearable PGHD over the 14 days prior to the PHQ-9 completion date.

*
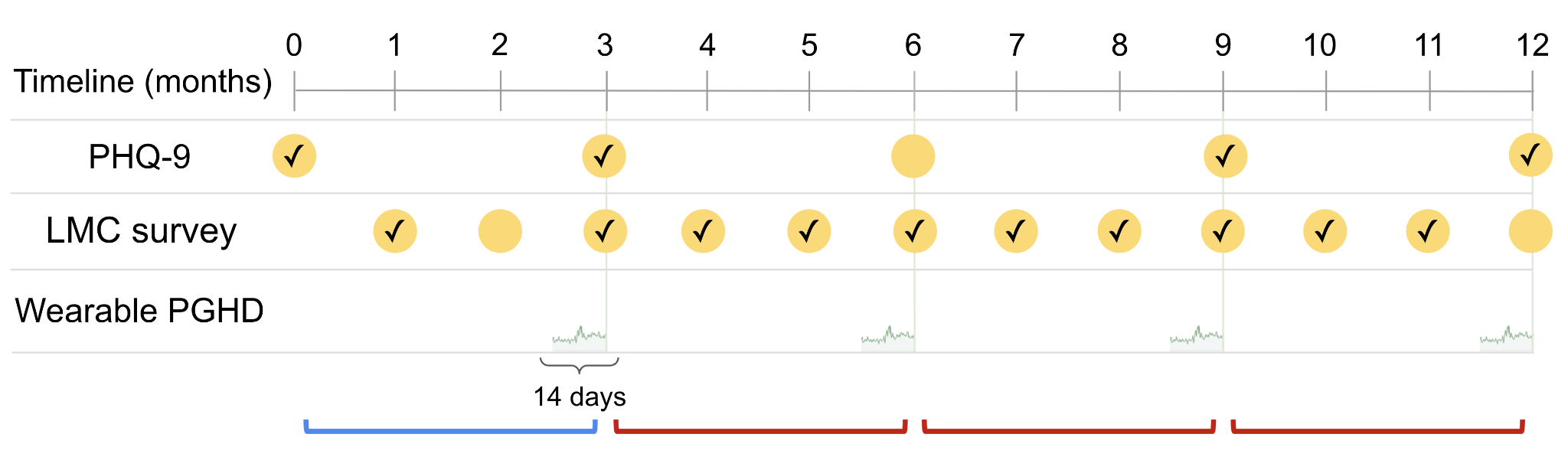
*

*Figure S1: Data collection timeline and survey completion criteria. Yellow circles represent data collection for a given month and data type, and check marks indicate completed surveys for an example participant. Wearable PGHD is collected in the 14 days prior to PHQ-9 completion date. Quarters marked in blue satisfy survey completion criteria, quarters marked in red do not. The first quarter (months 0-3), marked in blue, fulfills the selection criteria, as the initial and final PHQ-9 were completed at months 0 and 3, and the LMC survey was completed at month 3. The second quarter, marked in red, is not valid as the final PHQ-9 was not completed. The third quarter is not valid as the initial PHQ-9 was not completed. The fourth quarter is not valid as the LMC survey for the final month was not completed.*

### Data processing

###### Data filtering process

Of the approximately 25,000 initially screened participants for the DiSCover project[[2,4]](https://paperpile.com/c/FQjwBF/JHzyd+Zz9hy), 10,036 were enrolled into the study, and 9,961 passed the survey response quality control.

Then, based on survey completion, we kept participants who completed the PHQ-9 for at least two contiguous quarters, as well as the LMC survey for the same month as the second PHQ-9. The selection criteria based on survey completion are illustrated in the main manuscript. These requirements were set as we aim to study the evolution of depression severity, and recent lifestyle changes, such as changes in medication, are important factors in determining depression status.

Next, the participants were filtered based on the density of their available activity data in the two weeks matching the PHQ-9 recall period, according to standards proposed in literature[[5,6]](https://paperpile.com/c/FQjwBF/XSkp5+10jrZ). Participants were included if they had at least 4 valid days of activity data in each of the two weeks. A day is considered as “valid” if the participant wears the device for at least 10 hours that day.

For the phase 1 model, each sample in our dataset was defined as one observation of PHQ-9, one set of screener survey responses, one set of LMC survey responses, and wearable PGHD for a minimum of 8 and a maximum of 14 days. At this stage, we had samples from 4,036 unique participants, with 929 of the participants providing only one sample. Initial data exploration showed that the overall evolution of PHQ-9 scores was stable throughout the year when grouping by demographic variables, such as sex, age, race, and geographic location. Based on this observation, we treated each of the samples (scores) from the participants as independent from each other. Thus, we obtained a total of 10,866 samples from 4,036 unique participants.

Feature extraction and engineering was performed separately for survey responses and wearable PGHD, and is described in the following sections.

###### Survey responses

We split the survey response data into two categories: static and dynamic features. Features originating from the screener survey were considered as static as these features were not tracked for changes throughout the study. This includes features such as sex, race and comorbidities.

Lifestyle and medication changes (LMC) features were considered as dynamic, as we observed self-reported monthly changes in lifestyle and medication throughout the study. LMC features include changes in medication dosage or alcohol consumption habits in the past month.

###### Wearable PGHD

Wearable PGHD was collected using participants’ Fitbit devices throughout the study. In this work, we focused specifically on trends in step and sleep data.

Step and sleep data, initially provided at a minute-level granularity, were aggregated at day-level. These channels include the number of steps taken, number of active minutes (determined by step count per minute), number of minutes slept, number of minutes spent in bed, and the sleep efficiency score.

Three different approaches were used to generate feature sets. The first set was based on general statistical trends aggregated over three time windows, relative to PHQ-9 completion date (most recent): 4, 7, and 14 days. The statistical trends considered include mean, median, IQR, and range.

The second set of features was designed to observe changes in habits over the course of the 14 days. For two time windows – 7 days and 14 days – we fit linear regression models for various day-level channels, including number of minutes spent in bed and number of steps taken. For each fitted model, we used the resulting score, intercept and coefficient as features.

The third strategy consisted of defining threshold-based features. Specifically, we defined hypersomnia days (at least 10 hours of sleep), hyposomnia days (less than 5 hours of sleep), active days (at least 10,000 steps walked), and sedentary days (fewer than 5,000 steps walked). We counted the number and percentage of days for each of these categories, aggregated over two time windows: 7 and 14 days.

###### PHQ-9 depression severity labels

We aggregated PHQ-9 scores based on five predefined categories to measure the severity of depression in participants[[3]](https://paperpile.com/c/FQjwBF/q2lge). The category distribution is imbalanced, as expected in a random population sample. Table 2 presents PHQ-9 score categories, representing levels of depression severity, along with the number of samples in each category in our final dataset.

*Table S1: Description of the PHQ-9 score categories, representing depression severity levels, and the dataset category distribution.*

| **PHQ-9 total score** | **Depression severity** | **Number of samples** | **% of samples** |
| --- | --- | --- | --- |
| 0-4 | Minimal | 4202 | 38.7% |
| 5-9 | Mild | 3220 | 29.6% |
| 10-14 | Moderate | 1941 | 17.9% |
| 15-19 | Moderately severe | 981 | 9% |
| 20-27 | Severe | 522 | 4.8% |

### Modeling: phase 1c, categorisation of intermediate PHQ-9

The goal of the first phase model was to predict participants’ PHQ-9 score categories from sociodemographic, medical and wearable PGHD.

A common problem with wearable PGHD is inconsistent and missing data. Participants may choose not to wear their devices for various reasons, so we did not want to impose assumptions on their activity through imputation. This decision motivated our classification algorithm selection – the Light Gradient Boosting Machine (LightGBM) algorithm. LightGBM efficiently constructs ensembles of regression trees, and is able to handle sparse data. We specifically used the implementation with Dropouts Meet Multiple Additive Regression Trees (DART) boosting [[7]](https://paperpile.com/c/FQjwBF/PF0c) in order to be able to tune an additional dropout parameter to reduce overfitting to a given cohort.

As identified in the previous section, this is an imbalanced multi-class classification problem. To mitigate the effects of imbalanced classes, we performed sample stratification during training, hyperparameter tuning, and testing, so as to optimize performance across all categories.

We used a rigorous feature selection process in order to optimize model performance through dimensionality reduction. We removed highly correlated features, and used recursive feature elimination (RFE; [[8]](https://paperpile.com/c/FQjwBF/2eP5)) in order to eliminate features that had lower contributions to model performance.

Model performance was primarily measured using quadratic weighted Cohen’s Kappa. Weighted Kappa computes the level of agreement between the predicted and target values, using a distance-based weight penalty [[9]](https://paperpile.com/c/FQjwBF/O9w0p). Quadratic weighted Cohen’s Kappa uses a quadratic penalty in lieu of the traditional linear penalty based on the difference from the target value. The Kappa score ranges from -1 to 1, with a perfect score of 1 signifying full agreement, and -1 signifying a maximal distance between predicted and target values.

We also used adjacent accuracy (i.e. fraction of samples predicted at most one off from the target value), balanced accuracy and weighted F1-scores as secondary performance metrics.

#### Hyperparameter tuning

We performed randomized search 5-fold cross validation to tune the hyperparameters of our XGBoost model. We performed this procedure on feature subsets of survey response data, step data, sleep data, and combinations of each. The decision to select randomized search cross validation was made to optimize computational resources, as the performance of a complete grid search would require significantly more resources, at a marginal increase in model performance. We reported the performance metrics of the best tuned models with 95% confidence intervals across 5 training runs (5 outer shuffle splits).

### Modeling: phase 2c, prediction of longitudinal PHQ-9 change

In phase 2, we developed a model that predicts whether a person’s depression severity level has increased over a 3 month period, using PGHD and the intermediate PHQ-9 category classification generated by the phase 1 model. Figure 1 illustrates the data used in phase 1 and phase 2, to make a clear distinction between the goals of the two phases. In phase 1, we developed a model that predicts PHQ-9 score category using collected baseline and LMC survey data, as well as wearable PGHD data for a given month. Using this model, we generated intermediate monthly PHQ-9 score categories for SM1, denoting sample month 1, and SM2. In phase 2, we predicted an increase in PHQ-9 category using the person’s collected PHQ-9 results at the start of the 3 month period (SM0), generated predictions for intermediate PHQ-9 categories using the phase 1 model, and collected LMC survey responses and wearable PGHD for SM3. In addition to these data, we also used the screener survey responses as input features for both models, to control for sociodemographic factors.

To compute the target variable in each sample in the phase 2 model, we observed whether there was an increase in PHQ-9 category between SM0 and SM3. Each sample also consisted of input feature subsets that were generated from the following data:

- The starting PHQ-9 category at SM0, intermediate generated PHQ-9 categories at SM1 and SM2, as well as the generated probabilities of each PHQ-9 category for SM1 and SM2
- LMC survey responses collected at SM3
- Wearable PGHD collected over the 2 weeks prior to final PHQ-9 completion at SM3

In the following sections, we describe key performance metrics of the phase 2 model, as well as the feature and model selection processes, and we describe our participant-based validation strategy.

#### Performance metrics

The goal of the phase 2 model was to effectively identify and monitor participants whose depression level has increased, so naturally the primary metric used to evaluate model performance was the sensitivity score. Sensitivity, or the true positive rate, measures the proportion of positives that have been correctly identified. In practice, this means that we optimized our model in order to maximize the proportion of participants with increasing depression severity to be correctly identified.

We also wanted the model to be good at distinguishing between the two classes of participants, so we considered secondary performance metrics, including specificity and Area Under the Precision-Recall Curve (AUPRC)[[10]](https://paperpile.com/c/FQjwBF/ueMFn). We used AUPRC for model comparison, as it measures how well one model is able to distinguish between classes, especially when they are imbalanced. The baseline performance of the AUPRC metric is the fraction of samples with increased depression severity level, which corresponds to approximately 21% in our dataset.

Specificity allows us to consider the tradeoff between being able to correctly predict the target minority class (participants with increased depression severity), but not ignore the majority class of participants with stable or reduced depression severity. As we primarily targeted higher sensitivity performance we expected to have a lower specificity value, as in an eventual use case of our algorithm it would be better to perform an unnecessary check on people whose mental health is stable, rather than risk not checking in on participants whose mental health is declining.

#### Model construction and testing

As both the demographic and target class data are highly imbalanced in our dataset, we focused on building a robust model, such that it is not susceptible to data imbalance. As the goal of the model is to generalize to new participants, in the second phase, the model was fitted and tested on the same participant split as in the first phase, to avoid data leakage.

The model construction process took place in two phases: feature selection and model fitting. In the feature selection phase, we removed highly correlated features and performed recursive feature elimination with cross-validation [[8]](https://paperpile.com/c/FQjwBF/2eP5) on the largest subgroups of PGHD features: sleep data, step data, and static screener survey response data. This allowed us to identify a subset of features that was crucial to model performance, and eliminate some of the more noisy features. We then performed forward sequential feature selection using 5-fold cross validation on our training set. Forward sequential feature selection is a greedy algorithm that selects features based on which additional feature brings the most performance to the model, and has been previously used with success in longitudinal studies using digital biomarkers to predict mental health symptoms [[11]](https://paperpile.com/c/FQjwBF/FswEJ). In the model selection phase, in initial work [[12]](https://paperpile.com/c/FQjwBF/ZLZo), we assessed multiple models, but in the work presented here we used LightGBM with DART boosting, as in phase 1c. This implementation has been very performant in the initial work, so we proceeded with the same model.

We performed several experiments, based on the number of features selected in the forward sequential feature selection phase. Further details on this work are presented in [[12]](https://paperpile.com/c/FQjwBF/ZLZo).

#### Feature importance

The overall most important features were identified by a combination of two key metrics: ‘Gain’ importance and ‘split’ importance [[13,14]](https://paperpile.com/c/FQjwBF/Q32I7+atrco). Gain importance, also referred to as Gini importance is the improvement in accuracy brought by a feature to the branches it is on. Split importance, also called permutation importance, summarizes the number of times the feature is used in a model.

As LGBM is an ensemble tree-based learning model, features correspond to splitting nodes in each tree of the model. Gain importance for a given feature in the input feature set corresponds to the mean decrease in the feature node’s Gini impurity, proportional to the number of samples that the node splits (i.e. the number of samples have reached that node)[[15]](https://paperpile.com/c/FQjwBF/wywk8). This gives an absolute measure of feature importance.

Split importance was calculated as follows: model performance with a validation set (using 5-fold cross validation) was recorded as baseline performance. Then, for each feature in the input feature set, the rows of the feature column were randomly permuted in the input feature set, and model performance on the input dataset with the permuted column was computed. The difference in baseline performance and the latter performance was thus the split performance of that feature[[16]](https://paperpile.com/c/FQjwBF/w9kfa). Thus, we obtain a relative measure of feature importance.

Additionally, we recorded the total number of times that a given feature was selected during cross validation.

## Supplementary References

1. [Evidation Health’s DiSCover Program Completes Initial Enrollment, Releases Data from Largest U.S. Study of Chronic Pain [Internet]. 2019 [cited 2021 Jul 1]. Available from:](http://paperpile.com/b/FQjwBF/Phv8x) <https://evidation.com/news/evidation-healths-discover-program-completes-initial-enrollment-releases-data-from-largest-u-s-study-of-chronic-pain/>

2. [Lee JL, Cerrada CJ, Vang MKY, Scherer K, Tai C, Tran JLA, Juusola JL, Sang CN. The DiSCover project: Protocol and baseline characteristics of a decentralized digital study assessing chronic pain outcomes and behavioral data [Internet]. bioRxiv. medRxiv; 2021. [doi:](http://paperpile.com/b/FQjwBF/JHzyd) [10.1101/2021.07.14.21260523](http://dx.doi.org/10.1101/2021.07.14.21260523)[]](http://paperpile.com/b/FQjwBF/JHzyd)

3. [Kroenke K, Spitzer RL, Williams JB. The PHQ-9: validity of a brief depression severity measure. J Gen Intern Med [Internet] 2001 Sep;16(9):606–613. PMID:11556941](http://paperpile.com/b/FQjwBF/q2lge)

4. [Evidation Health’s DiSCover Program Completes Initial Enrollment, Releases Data from Largest U.S. Study of Chronic Pain [Internet]. 2019 [cited 2021 Jul 1]. Available from:](http://paperpile.com/b/FQjwBF/Zz9hy) <https://evidation.com/news/evidation-healths-discover-program-completes-initial-enrollment-releases-data-from-largest-u-s-study-of-chronic-pain/>

5. [Migueles JH, Cadenas-Sanchez C, Ekelund U, Delisle Nyström C, Mora-Gonzalez J, Löf M, Labayen I, Ruiz JR, Ortega FB. Accelerometer Data Collection and Processing Criteria to Assess Physical Activity and Other Outcomes: A Systematic Review and Practical Considerations. Sports Med [Internet] 2017 Sep;47(9):1821–1845. PMID:28303543](http://paperpile.com/b/FQjwBF/XSkp5)

6. [Tudor-Locke C, Camhi SM, Troiano RP. A catalog of rules, variables, and definitions applied to accelerometer data in the National Health and Nutrition Examination Survey, 2003-2006. Prev Chronic Dis [Internet] 2012 Jun 14;9:E113. PMID:22698174](http://paperpile.com/b/FQjwBF/10jrZ)

7. [Vinayak RK, Gilad-Bachrach R. DART: Dropouts meet Multiple Additive Regression Trees. Artificial Intelligence and Statistics [Internet] PMLR; 2015 [cited 2021 Sep 22]. p. 489–497. Available from:](http://paperpile.com/b/FQjwBF/PF0c) <https://proceedings.mlr.press/v38/korlakaivinayak15.html>

8. [Gene Selection for Cancer Classification using Support Vector Machines [Internet]. [cited 2021 Sep 22]. Available from:](http://paperpile.com/b/FQjwBF/2eP5) <https://paperpile.com/app/p/8f20f917-262b-065a-88b6-b65fb282ebaf>

9. [Fleiss JL, Cohen J. The Equivalence of Weighted Kappa and the Intraclass Correlation Coefficient as Measures of Reliability. Educ Psychol Meas [Internet] SAGE Publications Inc; 1973 Oct 1;33(3):613–619. [doi:](http://paperpile.com/b/FQjwBF/O9w0p) [10.1177/001316447303300309](http://dx.doi.org/10.1177/001316447303300309)[]](http://paperpile.com/b/FQjwBF/O9w0p)

10. [Saito T, Rehmsmeier M. The precision-recall plot is more informative than the ROC plot when evaluating binary classifiers on imbalanced datasets. PLoS One [Internet] 2015 Mar 4;10(3):e0118432. PMID:25738806](http://paperpile.com/b/FQjwBF/ueMFn)

11. [Sano A, Taylor S, McHill AW, Phillips AJK, Barger LK, Klerman E, Picard R. Identifying Objective Physiological Markers and Modifiable Behaviors for Self-Reported Stress and Mental Health Status Using Wearable Sensors and Mobile Phones: Observational Study. J Med Internet Res [Internet] JMIR Publications Inc.; 2018 Jun [cited 2021 Jun 10];20(6). PMID:29884610](http://paperpile.com/b/FQjwBF/FswEJ)

12. [Makhmutova M. Predicting changes in depression using person-generated health data [Internet]. 2021. Report No.: STUDENT. Available from:](http://paperpile.com/b/FQjwBF/ZLZo) <https://infoscience.epfl.ch/record/288435?&ln=en>

13. [Altmann A, Toloşi L, Sander O, Lengauer T. Permutation importance: a corrected feature importance measure. Bioinformatics [Internet] 2010 May 15;26(10):1340–1347. PMID:20385727](http://paperpile.com/b/FQjwBF/Q32I7)

14. [Breiman L. Random Forests. Mach Learn [Internet] Springer; 2001 Oct 1;45(1):5–32. [doi:](http://paperpile.com/b/FQjwBF/atrco) [10.1023/A:1010933404324](http://dx.doi.org/10.1023/A:1010933404324)[]](http://paperpile.com/b/FQjwBF/atrco)

15. [Perrier A. Feature Importance in Random Forests [Internet]. 2015 [cited 2021 Jun 15]. Available from:](http://paperpile.com/b/FQjwBF/wywk8) <https://alexisperrier.com/datascience/2015/08/27/feature-importance-random-forests-gini-accuracy.html>

16. [Raschka S. Feature Importance Permutation [Internet]. [cited 2021 Jun 15]. Available from:](http://paperpile.com/b/FQjwBF/w9kfa) <http://rasbt.github.io/mlxtend/user_guide/evaluate/feature_importance_permutation/>
