# Supplementary material for: Predicting Changes in Depression Severity Using the PSYCHE-D (Prediction of Severity Change-Depression) Model Involving Person-Generated Health Data: Longitudinal Case-Control Observational Study
Source: JMIR Mhealth Uhealth. 2022 Mar 25;10(3):e34148. doi: 10.2196/34148 (PMC8994145; doi:10.2196/34148)
Supplement: Multimedia Appendix 2 [file mhealth_v10i3e34148_app2.docx]

## **Multimedia Appendix 2.** Selected features.

## Note S1

The features selected for the final, best performing, hyperparameter tuned model in the phase 1c generation of intermediate depression severity labels:

| **Feature category** | **Feature description** |
| --- | --- |
| Screener survey (static) | Sex |
|  | Race/ethnicity (White, Black, Asian, Hispanic) |
|  | Birth year |
|  | Education level |
|  | Height |
|  | Weight |
|  | BMI |
|  | Pregnant |
|  | Experienced trauma resulting in injury (e.g. accident, fall) in the past year |
|  | Has health insurance |
|  | Has had sufficient money for basic living needs in the past month |
|  | Has received money or assistance from the government (e.g. food stamps, disability payments) in the past month |
|  | Number of individuals in the household |
|  | Comorbidity - Cancer |
|  | Comorbidity - Type 1 diabetes |
|  | Comorbidity - Gout |
|  | Comorbidity - Migraines |
|  | Comorbidity - Migraines - Average number of migraine days per month |
|  | Comorbidity - Migraines - Taking daily prescription medication for migraines |
|  | Comorbidity - Osteoporosis |
|  | Comorbidity - Neuropathic condition (number of conditions out of: fibromyalgia, peripheral neuropathic pain, central neuropathic pain) |
|  | Comorbidity - Arthritis (number of conditions out of: osteoarthritis, rheumatoid arthritis) |
| LMC (monthly) | Medication - Started a new medication |
|  | Medication - Stopped a medication |
|  | Medication - Changed dose of at least one medication |
|  | Medication - Do not use medication or non-medication therapy |
|  | Lifestyle - Began meditation or other relaxation techniques |
| Wearable PGHD | Number of days the participant has slept over 10 hours in the past 7 days |
|  | Range of the sleep start hour over the past 14 days |
|  | Mean ratio of the time spent asleep to the time spent in bed over the past 4 days |

We present the comparison between the gain importance and split importance for the features selected by the phase 1c model. The majority of lifestyle and medication changes (LMC) features tend to have a lower importance than screener features, and that screener features appear more frequently amongst different folds. This implies that static screener features are better predictors of absolute depression severity.

One of the three selected wearable PGHD features, the mean ratio of the time spent asleep to the time spent in bed over the past four days, is not visualised because its split importance is a magnitude higher than for the other features.

Overall, we note that the selected wearable PGHD features are absolute sleep-related statistics. Such statistics can be compared between participants, rather than within a given participant.


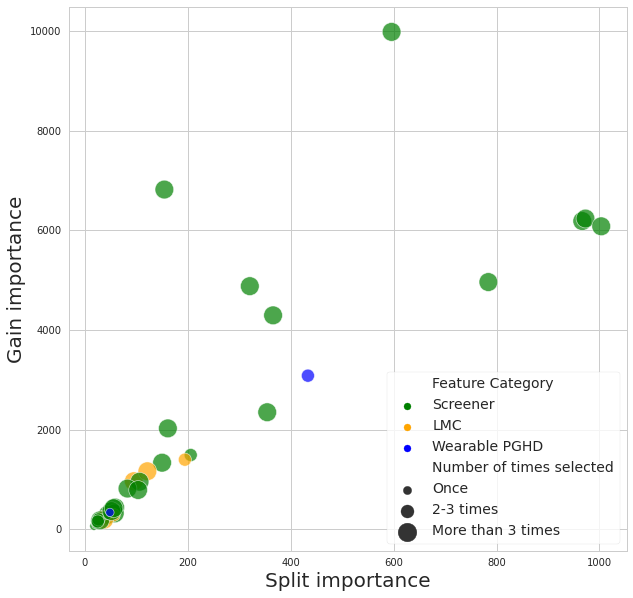


*Figure S1: Relative importance of selected features in the best performing phase 1c model, using participant-based splitting. Gain importance is plotted on the vertical axis and split importance on the horizontal axis. Each point represents the mean gain and split importance for a single feature, observed across 5-fold cross-validation. The size of the points reflects the number of times a feature has been selected over the random split (larger = more frequently selected), and color represents the type of feature (screener features = green, LMC features = yellow, wearable PGHD features = blue).*

## Note S2

The frequently selected features for the best performing phase 2c model with a participant-based splitting strategy are the following:

| **Feature category** | **Feature description** |
| --- | --- |
| PHQ-9 category | Collected baseline PHQ-9 category |
|  | Predicted PHQ-9 category at sample month 1 (2 months prior to prediction) |
| Screener survey (static) | Sex |
|  | Has health insurance |
| LMC (monthly) | Medication - Started a new medication |
|  | Medication - Stopped a medication |
|  | Medication - Changed dose of at least one medication |
|  | Medication - Stopped a non-medication therapy |
|  | Lifestyle - Began meditation or other relaxation techniques |
|  | Lifestyle - Reduced stress-inducing activities |
|  | Lifestyle - Reduced or stopped drinking alcohol |

As for Note S1, we also present the relative importance of these features, as measured by the metrics outlined in Multimedia Appendix 1.


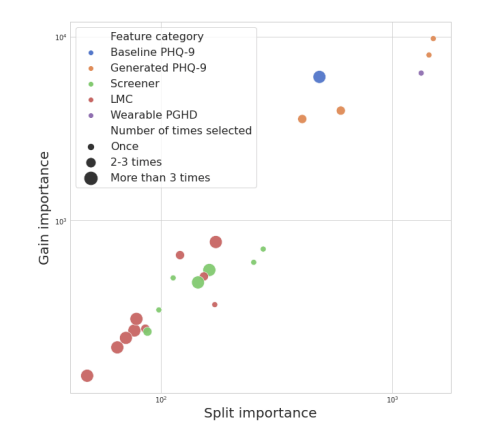


*Figure S2: Relative importance of selected features in the best performing phase 2c model in the combined pipeline. Gain importance is plotted on the vertical axis and split importance on the horizontal axis, each with a log scale. Each point represents the mean gain and split importance for a single feature, observed across 5-fold cross-validation. The size of the points reflects the number of times a feature has been selected for different splits (larger = more frequently selected), and color represents the type of feature (screener features = green, LMC features = red, wearable PGHD = purple, baseline PHQ-9 = blue, generated PHQ-9 features = orange).*
